# Infrequent false positive [^18^F]flutemetamol PET signal is resolved by combined histological assessment of neuritic and diffuse plaques

**DOI:** 10.1186/s13195-018-0387-6

**Published:** 2018-06-23

**Authors:** Milos D. Ikonomovic, Enrico R. Fantoni, Gill Farrar, Stephen Salloway

**Affiliations:** 1 Departments of Neurology and Psychiatry, University of Pittsburgh, Pittsburgh, PA USA; 2 Geriatric Research Education and Clinical Center, VA Pittsburgh Healthcare System, Pittsburgh, PA USA; 3 GE Healthcare Life Sciences, Amersham, UK; 4 Director of Neurology and the Memory and Aging Program at Butler Hospital in Providence, Providence, RI USA

**Keywords:** Flutemetamol, Amyloid, PET, Diffuse plaques, Neuritic plaques, Alzheimer’s disease

## Abstract

**Background:**

The performance of [^18^F]flutemetamol amyloid PET against histopathological standards of truth was the subject of our recent article in *Alzheimer’s & Dementia: Diagnosis, Assessment & Disease Monitoring* (2017;9:25–34).

**Main body:**

This viewpoint article addresses infrequently observed discordance between visual [^18^F]flutemetamol PET image readings and histopathology based solely on neuritic plaque assessment by CERAD criteria, which is resolved by assessing both neuritic and diffuse plaques and/or brain atrophy.

**Conclusion:**

[^18^F]flutemetamol PET signal corresponds predominantly to neuritic plaque pathology but is also influenced by the presence of diffuse plaques. This could allow for detection of diffuse amyloid deposits in the early stages of AD dementia, particularly in the striatum where diffuse amyloid is most commonly observed.

## Background

The advent of amyloid PET imaging has revolutionised clinical approaches to the differential diagnosis of dementia by enabling in vivo detection of fibrillar amyloid-β (Aβ) brain deposits [1]. Our recently published study [2] explored the performance of [^18^F]flutemetamol (GE Healthcare) [3, 4] amyloid PET image visual interpretation. Three standard of truth (SoT) measures were used to assess [^18^F]flutemetamol performance based on post-mortem neuropathology assessment. Both “Original” [5, 6] and “Modified” CERAD (Consortium to Establish a Registry for Alzheimer’s Disease) [7] SoT were based on assessing Bielschowsky silver stained neuritic plaques in, respectively, four and eight key regions. The CERAD criteria address only neuritic amyloid plaques, which have neuropathological diagnostic value in part due to the presence of dystrophic neurites and glial activation [8]. However, diffuse plaques may also contribute to the pathogenesis of Alzheimer’s disease (AD), as they are predominant in pathological ageing and in early disease stages [9], particularly in the striatum [10]. Hence, a third SoT assessment used anti-Aβ antibody (4G8) immunohistochemistry, enabling detection of both diffuse and neuritic Aβ plaques and their classification by Thal amyloid phasing [8, 11].

Of the 106 cases included in the original study [2], over 50% presented sparse-to-moderate neuritic Aβ plaques, which is at the threshold of amyloid positivity as per CERAD criteria [5]. This end-of-life population had mean age of 80.8 years and was 58% female. [^18^F]Flutemetamol PET was performed at a mean of 7.5 months before death. Seventy-eight subjects (73.6%) had a history of dementia and 53 (50%) had a clinical diagnosis of dementia due to AD [2].

**Fig. 1:**
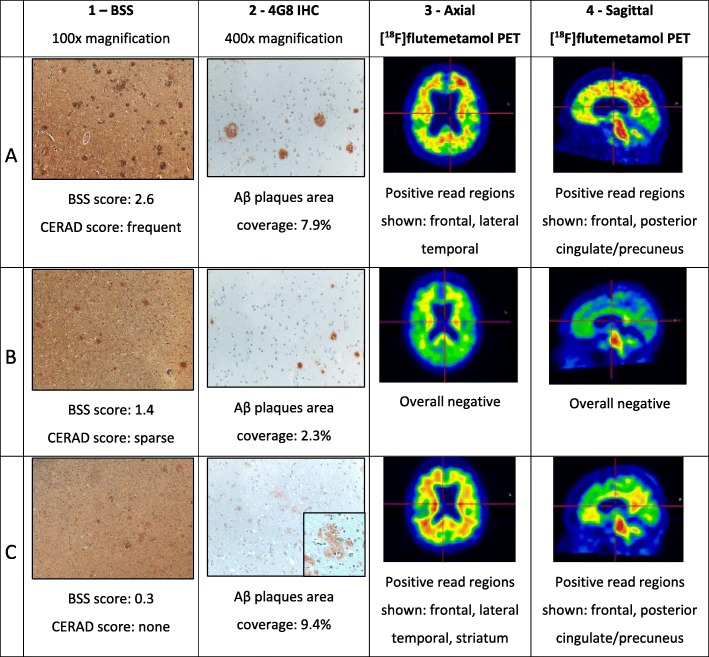
Case examples of fibrillar amyloid burden and corresponding [^18^F]flutemetamol PET images from subjects in [2, 7]. Histopathology samples from all eight cortical regions were in agreement. Examples are taken from frontal sections except for *row C*, *column 2* (*4G8 IHC*), a digital magnification showing diffuse amyloid plaques in the precuneus. *Column 1*: Bielschowsky silver staining (*BSS*) of neuritic plaques. *Column 2*: 4G8 Aβ immunohistochemistry (neuritic and diffuse Aβ plaques). *Column 3*: Axial [^18^F]flutemetamol PET. *Column 4*: Sagittal [^18^F]flutemetamol PET. *Row A*: True positive case (case 91 in [7]): 80-year-old male assessed as PET-positive by majority read (5/5 readers). *Row B*: True negative case (case 38 in [7]): 86-year-old male assessed as PET-negative by majority read (5/5 readers). *Row C*: False positive case (case 43 in [7]): 86-year-old female assessed as PET-positive by majority read (5/5 readers)

The assessment of [^18^F]flutemetamol performance relative to either CERAD method revealed a sensitivity and specificity of approximately 90%, indicating that neuritic Aβ plaques can explain the majority of the PET signal. This is supported by the correspondence between neocortical [^18^F]flutemetamol PET signal and neuritic Aβ plaques at autopsy (Fig. 1, rows A and B for case examples). When both neuritic and diffuse Aβ plaques were included in neuropathological assessments (i.e., according to 2012 National Institute on Ageing—Alzheimer’s Association (NIA-AA) guidelines [8]), the test reached 100% specificity, indicating that binding of [^18^F]flutemetamol to diffuse Aβ plaques additionally contributes to the amyloid PET signal.

## Main text

Notwithstanding the excellent concordance between [^18^F]flutemetamol PET reads and post-mortem amyloid pathology measures, it is useful to understand the discordant observations in ten out of 106 cases (three false positives (FPs), seven false negatives (FNs)) [2, 7]. Amyloid pathology in two FP cases was close to the CERAD sparse/moderate threshold in terms of neuritic plaque load. However, the additional [^18^F]flutemetamol PET signal from diffuse Aβ plaques resulted in positive PET reads. The third FP case (Fig. 1, row C) had very few neuritic Aβ plaques, consistent with negative CERAD assessments but unequivocally positive PET just 193 days before autopsy. A 0.3 CERAD score placed this case at the none/sparse boundary. However, the cortical Aβ plaques area percentage measured by immunohistochemistry was high at 9.3%. The case had β-amyloidosis Thal phase 4/5 (A3), neurofibrillary tangles Braak stage 3/6 (B2), and a clinical history of dementia. The neuropathology report also indicated significant Lewy body presence, leading to a Dementia with Lewy Bodies (DLB) diagnosis (case #43 [7]). However, using the 2012 NIA-AA diagnostic criteria this case would be considered to have intermediate AD neuropathology. These discordant cases exemplify how the 2012 NIA-AA AD diagnostic guideline update [8] including both neuritic and diffuse Aβ plaques can reclassify apparent false PET-positive into true PET-positive cases, slightly increasing [^18^F]flutemetamol’s specificity.

The seven FN reads reducing sensitivity to approximately 90% were due primarily to the presence of brain tissue atrophy. In this study, use of structural images in support of PET image interpretation was at the discretion of image readers [2]. Thus, when CT or MRI were not used, brain atrophy was not detected by the readers. Additionally, a couple of PET images had suboptimal signal-to-noise ratios or were subject to soft reconstruction, resulting in additional FN image reads. These factors lead to majority negative PET reads in cases with positive pathology, particularly by original CERAD around the sparse/moderate pathology border. When atrophy is suspected on PET scans, the corresponding CT or MR image should be used to understand its extent. Consequently, brain regions less susceptible to atrophy (e.g., inferior parietal and striatum) have been recommended for inclusion in the PET read user instructions [12].

In the context of AD pathology evaluation in living subjects, the use of amyloid PET to detect both neuritic and diffuse Aβ plaques enhances our ability to monitor the continuum of pathology progression. Diffuse plaques also contain Aβ fibrils [13] and may represent the initial phase of amyloid deposition [11, 14].

One region where diffuse Aβ plaques are the primary form of amyloid pathology is the striatum, an area which is a robust read region for [^18^F]flutemetamol [12] as well as showing a strong PET signal with [^11^C]PIB [15, 16]. In a study reported by Beach et al. [14], [^18^F]flutemetamol PET signal in the striatum had a high specificity (100%) when compared to striatal pathology as SoT. The lower sensitivity (83–87%), however, is consistent with the idea that the PET ligand’s binding to diffuse Aβ plaques is lower than to denser amyloid fibrils in neuritic Aβ plaques. The threshold for striatal amyloid positivity is implicated in Thal phase 3, which has been reported to correspond to clinical transition from cognitively normal to AD dementia [11]. This could contribute to utility of amyloid PET imaging in facilitating patient selection for clinical trials and for future amyloid-targeted therapies. Further support for the role of the striatum in early disease stages also comes from studies of Presenilin-1 mutation carriers, which showed this is the first region to demonstrate amyloid deposition in familial AD [15, 16].

## Conclusions

The research described here summarises the understanding of [^18^F]Flutemetamol PET performance metrics. This reinforces the high accuracy of this PET tracer and addresses the importance of pathology-based SoT methodologies and the nuances of PET image reading.

## Abbreviations

[^11^C]PIB: [^11^C]Pittsburgh Compound B
AD: Alzheimer’s disease
Aβ: β-Amyloid
BSS: Bielschowsky silver staining
CERAD: Consortium to Establish a Registry for Alzheimer’s Disease
DLB: Dementia with Lewy bodies
FN: False negative
FP: False positive
IHC: Immunohistochemistry
NIA-AA: National Institute on Ageing—Alzheimer’s Association
PET: Positron emission tomography
SoT: Standard of truth
